# Rapid Replacement of Acinetobacter baumannii Strains Accompanied by Changes in Lipooligosaccharide Loci and Resistance Gene Repertoire

**DOI:** 10.1128/mBio.00356-19

**Published:** 2019-03-26

**Authors:** Mark D. Adams, Meredith S. Wright, James K. Karichu, Pratap Venepally, Derrick E. Fouts, Agnes P. Chan, Sandra S. Richter, Michael R. Jacobs, Robert A. Bonomo

**Affiliations:** a The J. Craig Venter Institute, La Jolla, California, USA; b Department of Laboratory Medicine, Cleveland Clinic, Cleveland, Ohio, USA; c The J. Craig Venter Institute, Rockville, Maryland, USA; d Department of Pathology, University Hospitals Cleveland Medical Center, Cleveland, Ohio, USA; e Department of Pathology, Case Western Reserve University, Cleveland, Ohio, USA; f Department of Medicine, Case Western Reserve University and CWRU-Cleveland VAMC Center for Antimicrobial Resistance and Epidemiology, Cleveland, Ohio, USA; g Department of Pharmacology, Case Western Reserve University and CWRU-Cleveland VAMC Center for Antimicrobial Resistance and Epidemiology, Cleveland, Ohio, USA; h Department of Molecular Biology and Microbiology, Case Western Reserve University and CWRU-Cleveland VAMC Center for Antimicrobial Resistance and Epidemiology, Cleveland, Ohio, USA; i Department of Biochemistry, Case Western Reserve University and CWRU-Cleveland VAMC Center for Antimicrobial Resistance and Epidemiology, Cleveland, Ohio, USA; j Center for Proteomics, Case Western Reserve University and CWRU-Cleveland VAMC Center for Antimicrobial Resistance and Epidemiology, Cleveland, Ohio, USA; k Louis Stokes Cleveland Department of Veterans Affairs Medical Center, Cleveland, Ohio, USA; Northern Arizona University; Northern Arizona University

**Keywords:** *Acinetobacter*, antibiotic resistance, genome analysis

## Abstract

The population structure of health care-associated pathogens reflects patterns of diversification, selection, and dispersal over time. Empirical data detailing the long-term population dynamics of nosocomial pathogens provide information about how pathogens adapt in the face of exposure to diverse antimicrobial agents and other host and environmental pressures and can inform infection control priorities. Extensive sequencing of clinical isolates from one hospital spanning a decade and a second hospital in the Cleveland, OH, metropolitan area over a 3-year time period provided high-resolution genomic analysis of the Acinetobacter baumannii metapopulation. Genomic analysis demonstrated an almost complete replacement of the predominant strain groups with a new, genetically distinct strain group during the study period. The new group, termed clade F, differs from other global clone 2 (GC2) strains of A. baumannii in several ways, including its antibiotic resistance and lipooligosaccharide biosynthesis genes. Clade F strains are part of a large phylogenetic group with broad geographic representation. Phylogenetic analysis of single-nucleotide variants in core genome regions showed that although the Cleveland strains are phylogenetically distinct from those isolated from other locations, extensive intermixing of strains from the two hospital systems was apparent, suggesting either substantial exchange of strains or a shared, but geographically restricted, external pool from which infectious isolates were drawn. These findings document the rapid evolution of A. baumannii strains in two hospitals, with replacement of the predominant clade by a new clade with altered lipooligosaccharide loci and resistance gene repertoires.

## Abstract (precis)

Multidrug-resistant (MDR) A. baumannii is a difficult-to-treat health care-associated pathogen. Knowing the resistance genes present in isolates causing infection aids in empirical treatment selection. Furthermore, knowledge of the genetic background can assist in tracking patterns of transmission to limit the spread of infections in hospitals. The appearance of a new genetic background in A. baumannii strains with a different set of resistance genes and cell surface structures suggests that strong selective pressures exist, even in highly MDR pathogens. Because the new strains have levels of antimicrobial resistance similar to those of the strains that were displaced, we hypothesize that other features, including host colonization and infection, may confer additional selective advantages and contribute to their increased prevalence.

## INTRODUCTION

Acinetobacter baumannii is generally considered to be a pathogen of the hospital environment, as community-acquired cases are rare in the United States (1–3). Before the early 2000s, A. baumannii infections were uncommon but readily treatable. Antimicrobial resistance has increased dramatically during the last 2 decades, with a majority of clinical isolates now being multidrug resistant (MDR), based on resistance to at least three classes of antibiotics (1), and extremely drug-resistant (XDR) isolates have been reported, which cannot be killed by any FDA-approved antimicrobial agents (4). As a result, considerable attention is focused upon reducing hospital-acquired A. baumannii infections and limiting patient-to-patient transmission (5).

Two large complex hospital systems (denoted hospital system A and hospital system B) serve the same metropolitan area in northeastern Ohio, and their main tertiary care hospitals are located within 1 mile of each other in Cleveland. Their physician networks are largely nonoverlapping, and there is limited patient flow between the two systems due to constraints of provider networks. We previously showed considerable genomic diversity of A. baumannii strains isolated in hospital system A from 2007 to 2008 (6). Most strains belonged to four major clades (clades A to D) that are subsets of the GC2 (global clone 2) lineage (7, 8), and a fifth clade belonged to multilocus sequence type (MLST) group ST79 (clade E). In the context of examining genome changes in sets of multiple isolates from individual patients obtained from 2007 to 2013, we found further support for five major clades, each of which exhibits significant strain-level variation in gene content (9).

In the present study, we expanded the genome survey from hospital system A to cover nearly a decade, from 2007 to 2016. This longitudinal sampling allowed us to examine how A. baumannii populations diverged, revealing a dramatic change with time in the predominant genetic background of strains causing infections. In addition, we sequenced 70 A. baumannii strains isolated at hospital system B between 2012 and 2015. We sought to address whether these two hospital systems had distinct A. baumannii populations and, if not, to what extent the populations in the two health systems overlapped. By comparing strains from the two hospital systems, we tested the hypothesis that the hospital is the primary site of pathogen transmission. If true, we would expect closer relationships among isolates within each hospital system than among those between systems. Remarkably, we found an extensive overlap of strains in the two systems, with limited clustering of very closely related strains, suggesting a reservoir of strains outside these hospital environments.

## RESULTS

**FIG 1:**
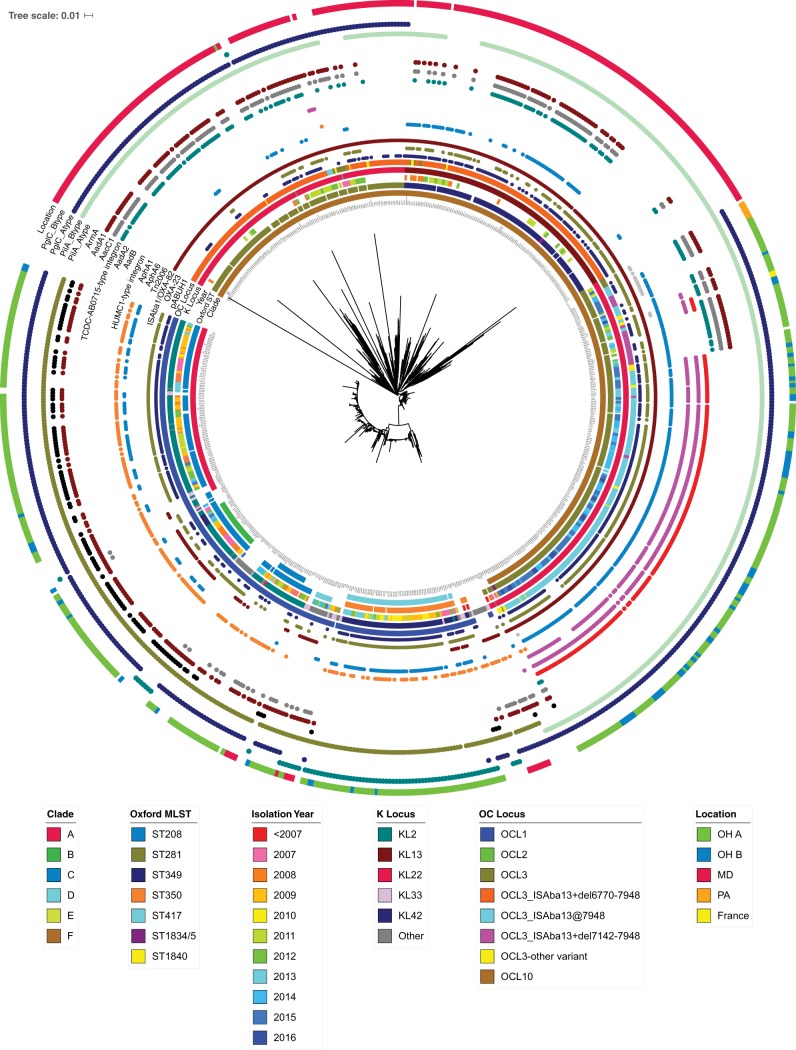
Phylogenetic tree of A. baumannii global clone 2 isolates. A phylogenetic tree was constructed using a core set of SNVs present in GC2 genomes. The rings of annotation extending outward from the inner ring depict clade, Oxford MLST type; year of isolation; K locus; OC locus; the presence of a pABUH1-like plasmid; OXA-23; the IS*Aba*1-*bla*_OXA_ insertion; Tn*2006*; AphA6; AphA1; the HUMC1-type integron; AadB; AadA2; the TCDC-AB7015-type integron; AacC1; AadA1; ArmA; two alternative PilA alleles, A-type PilA (GenBank accession number WP_000993717.1) and B-type PilA (GenBank accession number WP_000993718.1); two alternative PglC alleles, A-type PglC (GenBank accession number WP_000826956.1) and B-type PglC (GenBank accession number WP_000977704.1); and isolation location.

To place the metropolitan Cleveland isolates into a broader phylogenetic perspective, we combined 371 hospital system A and 70 hospital system B genome sequences with 354 additional genome sequences available in GenBank (see Materials and Methods). Phylogenetic analysis was performed with both the full set of 795 genomes and the 711 genomes that correspond to GC2 isolates, since these were the most common among the Cleveland strains (Fig. 1; see also Fig. S1 and Fig. S2 in the supplemental material). The GC2 isolates clustered into five major clades, four of which corresponded to those described previously (6). More than 90% of the Cleveland isolates belonged to GC2. Non-GC2 genomes were more diverse based on gene content and the extent of homologous recombination and tended to have fewer antibiotic resistance genes (Fig. S1).

10.1128/mBio.00356-19.1 FIG S1 Phylogenetic tree of all A. baumannii strains from this study. The rings of annotation extending outward from the inner ring depict clade; OC locus; Pasteur MLST; Oxford MLST; genes encoding StrA, OXA-23, the IS*Aba*1-*bla*_OXA_ insertion, AphA6, AphA1, AadB, AadA2, AacC1, AadA1, A-type PilA (GenBank accession number WP_000993717.1), B-type PilA (GenBank accession number WP_000993718.1), A-type PglC (GenBank accession number WP_000826956.1), B-type PglC (GenBank accession number WP_000977704.1), and C-type PglC (GenBank accession number WP_000919783.1); and isolation location. Download FIG S1, PDF file, 0.7 MB. Copyright © 2019 Adams et al. 2019 Adams et al. This content is distributed under the terms of the Creative Commons Attribution 4.0 International license.

10.1128/mBio.00356-19.2 FIG S2 Phylogenetic tree of all clade F A. baumannii strains from this study. The rings of annotation extending outward from the inner ring depict Oxford MLST; year of isolation; K locus; OC locus; the presence of a pABUH1-like plasmid; genes encoding OXA-23, the IS*Aba*1-*bla*_OXA_ insertion, AphA6, AphA1, the HUMC1-type integron, AadB, AadA2, the TCDC-AB0715-type integron, AadC1, AadA1, ArmA, B-type PilA (GenBank accession number WP_000993718.1), and B-type PglC (GenBank accession number WP_000977704.1); and isolation location. Download FIG S2, PDF file, 1.4 MB. Copyright © 2019 Adams et al. 2019 Adams et al. This content is distributed under the terms of the Creative Commons Attribution 4.0 International license.

### Emergence of a new GC2 group, clade F.

All five major GC2 clades belong to MLST group ST2 (Pasteur scheme [10]). The largest clade in the tree, termed clade F, includes 173 Cleveland strains, representing 32% of the isolates from the two hospital systems, and 250 genomes downloaded from GenBank, including 232 from a comparable health care system in Baltimore, MD (11) (Fig. S2). Clade F corresponds to two strain types in the Oxford (12) MLST scheme: ST281 and a single-locus variant, ST349. The ST349 and ST281 genomes are in separate phylogenetic groups.

The Cleveland and Baltimore clade F isolates are located in distinct branches of the tree, with the exception of UH7007, which was isolated 4 years before clade F strains began to appear more broadly. The few isolates from Pittsburgh (13) and Chicago (14) are located outside the Cleveland group on the tree. Considerable diversity of gene content was observed among clade F isolates, particularly those from Baltimore (Fig. 1). A few strains were isolated from distant geographic locations, including HUMC1, isolated in 2009 in Los Angeles, CA (15); ABIsac_ColiS and ABIsac_ColiR, isolated in France in 2011 (16, 17); and ORAB10, from Oregon in 2013 (18). Most clade F strains were isolated after 2011; however, this clade is likely much older than that based on the phylogenetic distances from other GC2 genomes and based on the variability in genome content, particularly among the Baltimore isolates. An average of 690 ± 110 single-nucleotide variant (SNV) differences separate core genome regions of clade F genomes from those in clades A to D, compared to generally fewer than 100 SNV differences within clades A to D.

### Temporal patterns of A. baumannii.

**FIG 2:**
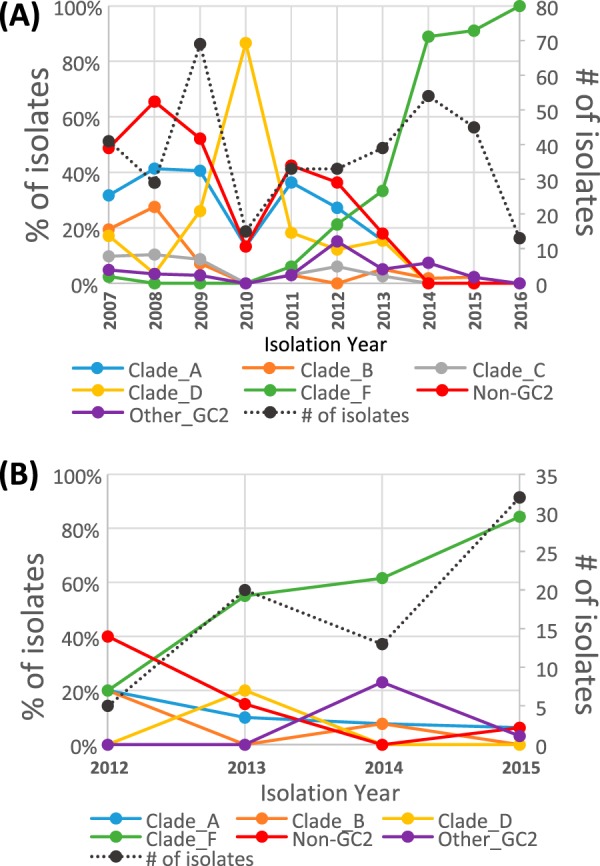
Change in clade abundance over time. The proportions of A. baumannii isolates from hospital system A (A) and hospital system B (B) belonging to each major clade are shown, grouped by the year in which they were isolated. The number of isolates per year is shown as a black dotted line, with units provided on the secondar*y* axis (*n* = 443 for UH strains and *n* = 70 for CC strains).

Before 2013, the majority of strains from hospital system A originated from five distinct clades (Fig. S1A to E). The frequency of clade F began to rise in 2014 (Fig. 2). By the end of 2015, most of the strains belonged to clade F. This demonstrates a remarkable decrease in overall genetic diversity and a major restructuring of the population.

Extensive overlap was evident in the phylogenetic placement for hospital system A and B strains isolated between 2012 and 2016. Each of the major clades contained genomes from isolates from both hospital systems. Approximately 80% of the strains isolated in 2014 to 2016 at both systems belonged to clade F. The average number of SNVs between clade F strains did not differ either within the hospital system genome sets or between the two sets (15 ± 7). The shared locations of insertion sequence (IS) elements were concordant with the SNV tree in supporting intermixing of strains between the two hospital systems (data not shown).

### Clade F strains have very limited hospital-specific clustering.

The small number of SNV differences between strains makes precise inference of phylogenetic relationships difficult. However, two broad patterns were apparent in the distribution of Cleveland strains across the phylogenetic tree. First, extensive intermixing of genomes between the two hospital systems was revealed, indicating that strains diverged before entry into the hospital systems. This is most apparent in clade F, which was expected because it had the most isolates collected over similar time periods in both hospital systems. Second, there were small clusters of strains adjacent to one another on the tree that may represent local outbreaks within one hospital system. Sufficient information about patient locations was not available to support a more rigorous analysis of epidemiological patterns.

### Antibiotic resistance gene features of clade F strains.

Overall, the antimicrobial resistance phenotypes of Cleveland strains from clade F are similar to those from other GC2 strains (Table 1). The antimicrobial resistance gene content of clade F strains differed in many significant ways from those of other GC2 isolates. The Tn*1548* genomic island carries the *armA* gene, which confers resistance to most aminoglycoside antibiotics and is common in isolates from clades A and B. Tn*1548* was absent from clade F strains, and the genome sequence where it is typically inserted adjacent to the homolog of ACICU_02399 was intact in these strains, with no evidence of an IS*26* or transposon insertion at that location. Aminoglycoside resistance in clade F strains from Cleveland is likely to be mediated by genes carried in one of two alternative integron structures, as described below, and by a plasmid-borne *aphA6* gene.

**TABLE 1:** Antibiotic susceptibility phenotypes of A. baumannii isolates^*a*^

Clade	Amp/ sulbactam		Cefepime		Ceftazidime		Imipenem		Meropenem		Amikacin		Gentamicin		Tobramycin	
	No. of strains	% non-S strains	No. of strains	% non-S strains	No. of strains	% non-S strains	No. of strains	% non-S strains	No. of strains	% non-S strains	No. of strains	% non-S strains	No. of strains	% non-S strains	No. of strains	% non-S strains
A	82	96.3	82	97.6	82	100.0	56	100.0	82	97.6	54	79.6	82	96.3	82	87.8
B	23	73.9	25	100.0	25	100.0	18	83.3	25	100.0	18	77.8	25	96.0	25	88.0
C	15	93.3	16	100.0	16	100.0	13	53.8	16	43.8	11	18.2	16	93.8	16	25.0
D	56	75.0	56	98.2	55	98.2	36	94.4	55	94.5	42	78.6	56	96.4	55	96.4
F	169	81.7	155	95.5	157	100.0	155	99.4	169	99.4	161	95.0	169	93.5	167	80.8
Other GC2	22	90.9	21	100.0	21	100.0	19	89.5	22	90.9	20	80.0	22	100.0	22	86.4
Non- GC2	43	60.5	44	81.8	44	65.9	33	90.9	37	89.2	33	54.5	44	56.8	44	54.5

a Shown are the numbers of strains in each group for which susceptibility data were available for the indicated antimicrobial agent. non-S, nonsusceptible; Amp, ampicillin.

Most Cleveland clade F strains carried the *bla*_OXA-23_ gene on a pACICU2-like plasmid (19); however, it appears that the plasmid was lost from several isolates distributed throughout the clade F group. Upregulation of the endogenous OXA-51 family carbapenemase gene *bla*_OXA-82_ by insertion of an upstream IS*Aba*1 element that carries a strong outward-facing promoter can also lead to high-level resistance to imipenem and meropenem (20, 21). All but two clade F strains had the IS*Aba*1-*bla*_OXA-82_ structure, which may make the plasmid-borne *bla*_OXA-23_ gene redundant in certain growth environments. Most clade B isolates carried the IS*Aba*1-*bla*_OXA_ structure, along with several strains in clades C and D. No clear difference was seen in the patterns of susceptibility to carbapenem drugs for isolates with IS*Aba*1-*bla*_OXA-82_ compared with isolates with *bla*_OXA-23_ or both genes. Fifteen clade F strains carry Tn*2006*, which harbors *bla*_OXA-23_ flanked by IS*Aba*1 elements (22, 23). The genomes with Tn*2006* are located at several distinct locations on the phylogenetic tree, suggesting multiple independent acquisitions.

The pACICU2-like plasmid that was the most common in clade F isolates from Cleveland is closely related to pABUH1 that carries both the *bla*_OXA-23_ gene in Tn*2008* and *aphA6* in Tn*aphA6* (7, 24). In genomes from Cleveland isolates, the absence of *bla*_OXA-23_ generally correlated with loss of the plasmid. Loss of the plasmid did not always result in loss of Tn*aphA6*, however, suggesting that this element can mobilize to other genomic locations. Clade F isolates have about a dozen copies of IS*Aba*125, making it impossible to precisely discern the location(s) of the Tn*aphA6* transposon in these draft genome sequences. There was more variability in plasmid sequences represented in the assemblies of genomes among the Baltimore isolates, and Tn*2008* and Tn*aphA6* could not be linked to the plasmid in many cases.

**FIG 3:**
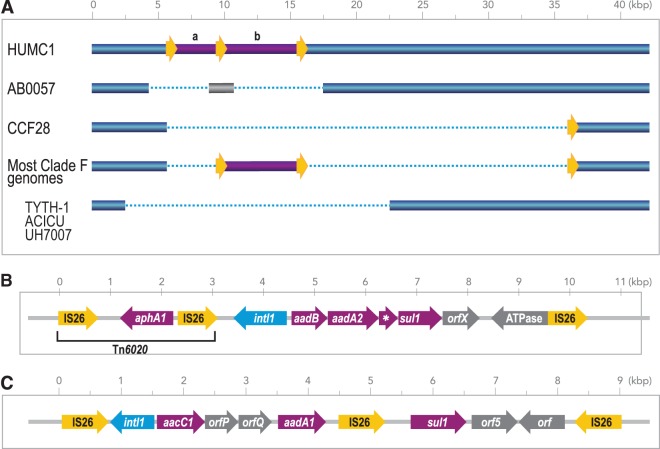
Integron structures in clade F strains. (A) A complex transposon structure was inserted into the HUMC1 genome at coordinates 1513187 to 1528810 (GenBank accession number LQRQ01000007.1). A partial deletion of this region was found at the corresponding location in the GC1 strain AB0057 and in other GC2 strains, including TYTH-1, ACICU, and the clade F strain UH7007. Most clade F genomes carried only the integron (labeled “b”) and not the Tn*6020* segment (labeled “a”) from HUMC1. Yellow arrows indicate copies of IS*26*. (B) Gene diagram corresponding to the region in purple in panel A. (C) The complex transposon structure from ABUH585 and other clade F genomes. This region is similar to the integron present in the TCDC-AB0715 genome at bases 274173 to 281008 under GenBank accession number CP002522.2. Resistance genes are in purple. The * gene in panel B is *qacE*Δ*1*.

Class 1 integrons carrying a range of antibiotic resistance genes are common in MDR A. baumannii (25). Clade F strains from Cleveland carry one of two distinct class 1 integrons. One integron carries a resistance gene cassette that has not been previously described in A. baumannii (Fig. 3A and B). This cassette includes the *aadB* gene encoding aminoglycoside (2′)-adenyltransferase ANT(2″)-Ia and *aadA2* encoding streptomycin 3″-adenylyltransferase. A search by BLAST of the NCBI database revealed that a nearly identical integron sequence was present in Proteus mirabilis strain AR_0059 (GenBank accession number CP020052.1); several strains of Escherichia coli isolated from cow, pig, and chicken; and Salmonella enterica subsp. *enterica* (e.g., GenBank accession number CP014658.1). It appears that the integron was mobilized by a pair of IS*26* elements in direct-repeat orientation. In strain HUMC1, an IS*26*-flanked Tn*6020* element carrying *aphA1* was located immediately adjacent to the integron. However, Tn*6020* was not found in any other clade F strains. Previously, the *aadB* gene has been found most often associated with the small plasmid pRAY in A. baumannii (7). A few clade F strains have *aadB* in a different genomic context. Strains without *aadB* were more likely to be susceptible to tobramycin (data not shown).

The second integron is closely related to a sequence from TCDC-AB0715 (26) (GenBank accession number CP002522.2), except that the *qacE*Δ gene is interrupted by an IS*26* element (Fig. 3C). This structure includes the *aacC1* and *aadA1* genes, encoding aminoglycoside *N*(3′)-acetyltransferase and streptomycin 3″-adenylyltransferase, respectively. CCF48, CCF75, ABUH628, and HUMC1 appear to contain both integrons, whereas all other clade F genomes contain only one of them. The novel integron structure (Fig. 3B) is largely restricted to Cleveland isolates, whereas the TCDC-AB0715-like integron was present in more than half of the genomes of Baltimore isolates.

Most MDR GC2 A. baumannii genomes described to date have an AbaGRI1-type resistance island, also termed Tn*6166*, inserted into the *comM* gene (27–29). In contrast, most clade F strains lack an insertion at that location. CCF66 and CCF31 have an ∼20-kb island that is very similar to Tn*6166* and includes *strAB* and a *tetA* gene (30). ABUH773 and eight other strains have a related structure at this location that lacks about 8 kb at the 3′ end of the element, including the *strAB* genes. In addition to resistance to antibiotic treatment, A. baumannii is subjected to selective pressure in the context of competition for resources with its host, immune evasion, and survival in the hospital environment. Genes involved in the host response and virulence include those related to motility, cell surface glycosylation, attachment to biotic and abiotic surfaces, micronutrient acquisition, and secretion systems (3). Genes related to these traits are also variable among clade F strains.

**FIG 4:**
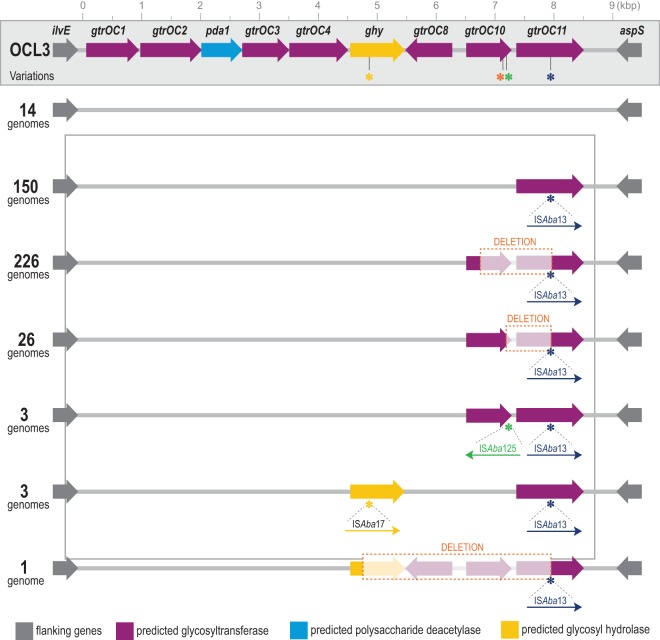
Variation in the OCL locus among clade F strains. The inferred genetic structure of the OCL locus is shown, with variants present in different genomes. The OCL3 locus from GenBank accession number KC118540.6 is shown. Asterisks denote the locations of IS element insertions in a subset of genomes. The OCL3 variants found among clade F genomes are depicted, along with the number of genomes in which the variant was observed.

Two genetic loci carry genes whose products determine the structure of the lipooligosaccharide (LOS) structures on the cell surface (31, 32). Clade F strains were distinct from other GC2 strains by expressing the KL22 or KL13 variant at the K locus and an OCL3-like variant at the OC locus (Fig. 1). Fourteen clade F genomes have an intact OCL3 locus, but all other clade F strains carried one of several variants. An IS*Aba*13 element interrupted the last gene in the cluster (*gtrOC11*), which encodes a glycosyltransferase (Fig. 4). Many clade F strains had additional modifications to the OCL3 locus, including deletions (adjacent to the IS*Aba*13 insertion site) and insertions of IS*Aba*125 and IS*Aba*17. Kenyon et al. showed a similar pattern of IS element disruption of the OCL1 locus in GC2 isolates from Australian hospitals that resulted in truncated LOS structures (33). KL13 strains belong to ST349, while KL22 strains belong to ST281 in the Oxford MLST scheme.

Most A. baumannii strains express a type IV secretion system comprised of a complex cell surface pilus structure. The structure is encoded by multiple genes, and it has been noted that there is variation in the critical PilA structural pilin protein (34). Most GC2 strains carried a *pilA* gene homologous to ACICU_03380. Clade F strains harbored a different PilA family gene at this position, typified by the protein under GenBank accession number ETR04673.1 from UH7007. The two encoded proteins shared only 65% amino acid similarity. Interestingly, the gene replacement event in clade F strains also resulted in deletion of the adjacent gene downstream of the *pilA*-like gene, ACICU_03379, which encodes a Wzy_C (O-antigen ligase) domain-containing protein. The loss of this gene in clade F strains may result in additional differences in cell surface structure compared with other GC2 A. baumannii strains.

We also used a comprehensive pangenome analysis of the Cleveland isolates to identify broader patterns of genes that are shared and distinct among the clades. A neighbor-joining tree created using a distance matrix based on the number of shared genes largely supported the clades identified in the SNV tree (Fig. S3A). Fifty-three genes were specific to clade F, and 19 genes were present in all other clades but were missing from clade F. Most of these genes were annotated as encoding hypothetical proteins and may represent poorly annotated prophage. Within clade F, there was little correlation between the order of strains in the SNV tree and the shared-genes tree (Fig. S3B), indicating that there were recent changes in gene content, such as independent losses of the pACICU2-like plasmid and IS*26-*associated deletions. The clade A genomes comprised several subgroups in the pangenome analysis. One group of those clade A groups lacked the pACICU2-like plasmid and the *bla*_OXA-23_ gene. Clade D genomes had 109 clade-specific genes and lacked 51 genes that were present in all other clades. Clade D genomes encoded the KL42 capsular K locus cassette, and several of the differentially present genes were within this locus. Several metabolic enzymes were also encoded among the clade D-specific genes.

10.1128/mBio.00356-19.3 FIG S3 Pangenome shared gene content tree. (A) The number of genes shared by each strain was expressed as a distance matrix and used to create a neighbor-joining tree. The outer circles represent clade and hospital of origin. (B) Comparison of the order of strains in the SNV tree from Fig. 1 with the order in the gene content tree from panel A. Each point represents one genome, color-coded by clade. Download FIG S3, PDF file, 0.4 MB. Copyright © 2019 Adams et al. 2019 Adams et al. This content is distributed under the terms of the Creative Commons Attribution 4.0 International license.

## DISCUSSION

Long-term population dynamics reflect the cumulative impact of dispersal, mutation, introgression of new genetic backgrounds, diversification through lateral gene transfer or homologous recombination, and selection in the local environment. Here we show that the predominant genetic background of A. baumannii strains shifted in a similar way during the course of several months, resulting in very closely related strains becoming predominant in two different hospital systems serving the same geographical area. Interleaving of the genomes from the two hospital systems in the phylogenetic tree and analysis of shared genomic features suggest multiple introductions of closely related but distinct A. baumannii strains with small local expansions.

The emergence and replacement of previous genetic backgrounds in both systems spanning the same time period could be due to several factors that may have contributed to shaping the set of strains causing infections. First, a common source of new strain backgrounds might exist in extended care facilities that exchange patients with both hospital systems (35, 36). The involvement of asymptomatic carriage and transmission of the Gram-negative pathogen Klebsiella pneumoniae was recently hypothesized as an explanation of genome variation observed in a large survey of isolates from multiple hospitals (37). A reservoir for A. baumannii outside health care settings has never been definitively demonstrated, and community-acquired infections are rare in the United States (38).

Second, the pattern of strains in Cleveland could reflect a broader shift in A. baumannii strains in the United States. Systematic genome-based surveys of A. baumannii strains do not presently exist in the United States, and so it is not possible to determine how representative the isolates from these Cleveland hospitals are. As shown in Fig. 1, isolates from Chicago, Baltimore, and Pittsburgh are all located in separate phylogenetic branches from the Cleveland isolates, suggesting geographical separation of the Cleveland isolates. There are, however, three non-Cleveland strains that cluster with the genomes from Cleveland in the core genome phylogeny: two strains isolated from the same patient in France (17) and one from Oregon. It seems likely that as additional isolates are sampled, there will be more examples of genomes that share a recent common ancestor with the Cleveland strains. This study along with those described above show that SNVs in the nonrecombinant core genome are only one view of shared genetic content. A full picture of relationships between strains needs to encompass SNVs, mobile elements, and homologous recombination events. To highlight this, the two genomes from France have only 3 IS*Aba*1 copies, compared to 22 to 29 for the Cleveland isolates.

Finally, the clade F strains were not simply added to the population in the Cleveland hospital systems; over the course of about a year, they displaced the previously dominant lineages. The overall levels of antibiotic resistance are similar in strains from clade F and from other lineages (Table 1). Thus, while it is possible that antibiotic pressure contributed to the rise of clade F, we also hypothesize that these strains have some advantage in either survival or transmissibility. Harding et al. have summarized the current knowledge of virulence mechanisms in A. baumannii (3). Many of the mechanisms that they highlight are conserved across all A. baumannii genomes, including the type I and II secretion systems; the Ata protein, which is important for adherence to host surfaces; and the type IV pilus. However, several of the genes highlighted in the review by Harding et al. are absent or variable in the clade F genomes. The presence of a putative alternative major pilus protein, PilA, in the clade F genomes may indicate a functional difference of this structure that is important for twitching motility and adherence to epithelial cells (39, 40). The three-dimensional structures of three variant PilA proteins were recently described (34), providing insight into functional differences among these proteins. The *pilA* allele in clade F genomes is distinct from all of those, with <80% amino acid identity of the encoded protein.

Lipopolysaccharide, lipooligosaccharide, and capsule structures contribute to evasion of innate immune responses in several pathogens, including Escherichia coli (41). Capsule replacement has also been suggested to have contributed to the success of the ST258 lineage of Klebsiella pneumoniae (42). We also found an enrichment in variants affecting cell surface structures among longitudinally sampled A. baumannii strains from individual patients (9). Most GC2 A. baumannii strains carry the OCL1 locus, whereas clade F strains carry several variants of the OCL3 locus. These variants are characterized by multiple intragenic IS element insertions and deletions, suggesting the loss of some glycosyltransferase activities. Multiple novel variants of the phosphoglycosyltransferase gene *pglC* that catalyzes the initial step of capsule synthesis (43) were also found among the GC2 genomes. The functional impact of variation at these loci bears further investigation.

Among genome features of the clade F strains, relatively few are unique to the Cleveland isolates. The HUMC1-type integron (Fig. 3B) is restricted to HUMC1 and the Cleveland isolates, among the genomes that have so far been described. This integron carries *aadB* and *aadA2*, which confer aminoglycoside resistance, and while this structure is novel, those genes are present in different genetic contexts in other A. baumannii genomes. The alternative *pilA* allele is shared by Cleveland and non-Cleveland clade F genomes, as are the OC and K locus variants.

A number of studies have used genomic approaches to enhance epidemiological studies of A. baumannii in hospital environments (44–46). We previously found that almost one-third of patients with multiple A. baumannii isolates were infected by more than one genetically distinct strain (9). Others have seen a similar diversity of cocirculating strains. Schultz et al. found multiple distinct lineages of carbapenem-resistant A. baumannii in a Vietnamese hospital and documented the transposon-mediated spread of a *bla*_OXA-23_ gene to new genetic backgrounds (47). These investigators also observed switching of the capsule locus genes, likely by homologous recombination in flanking regions. A yearlong survey of a German intensive care unit found that half of the A. baumannii isolates belonged to one of two clusters but that all others were genetically distinct from each other (48).

A somewhat different picture emerged in an analysis of 85 strains from Mexico (49). The Mexico strains had very few SNV differences compared to a broad set of reference genomes and thus appeared to be clonally related. However, they exhibited extensive variation in gene content, with scores of strain-specific genes and with few genomes highly similar in gene content. Chromosomal and plasmid-mediated mobile elements were a chief source of the variation in gene number, but the origins of the variable gene content could not be determined. Feng et al. characterized A. baumannii isolates from East Asia and also found strains that were very closely related in their core gene phylogeny despite being isolated in hospitals that are more than 2,500 km apart (50). Even closely related genomes had differences in mobile elements related to antibiotic resistance. A common theme of these studies and the current survey of A. baumannii genomes from Cleveland is that A. baumannii genomes exhibit a high degree of genomic variation and that apparently closely related strains can nonetheless differ in genome composition, leading to phenotypic differences.

Knowledge regarding mechanisms by which MDR pathogens are transmitted among and within hospitals is important for understanding how new resistance genes or virulent lineages disperse from local to global scales. The rapid shift in genetic background described here highlights the importance of genetic monitoring of strains causing infection as part of an ongoing effort to optimize treatment and prevention strategies. Extensive genic diversity is a hallmark of A. baumannii isolates. While the antimicrobial resistance genotypes are certainly an important component of this variation, the breadth of functions represented among genes that are acquired and lost suggests that other selective forces, such as host interaction and survival, also play a significant role in shaping the genome evolution of A. baumannii and that these areas are worthy of further exploration in developing an improved understanding of the basis for the clinical impact of this pathogen.

## MATERIALS AND METHODS

### Strains.

Clinical isolates of A. baumannii were archived by the clinical microbiology departments under protocols approved by institutional review boards at each institution. Isolates from hospital system A were collected between 2007 and 2016 and stored at −80°C. Samples were randomly selected for sequencing to span the time frame of collection. In cases where more than one strain was isolated from a patient, only one isolate was included in the analysis presented here.

Isolates from hospital system B were obtained from bloodstream infections during 2012 and 2016. Only meropenem-resistant strains were selected; these represented 51% of all A. baumannii bloodstream infections during this period. A total of 371 strains from hospital system A and 70 from hospital system B were included. Genome sequences for 344 of those strains were determined in this study, and the remainder have been reported previously (6, 9). Accession numbers for all genomes are available in Data Set S1 in the supplemental material.

10.1128/mBio.00356-19.4 DATA SET S1 Strain list with accession numbers and antimicrobial susceptibility information. Download Data Set S1, XLSX file, 0.1 MB. Copyright © 2019 Adams et al. 2019 Adams et al. This content is distributed under the terms of the Creative Commons Attribution 4.0 International license.

### Genome sequencing, annotation, and comparative analysis.

DNA was isolated using mechanical lysis with glass beads (51). Libraries were prepared for sequencing using NexteraXT kits and sequenced on an Illumina NextSeq 500 DNA sequencer as paired-end, 150-base reads. Read sets were assembled using velvet (52) and submitted for annotation to the Prokaryotic Genome Annotation Pipeline (PGAP) at the National Center for Biotechnology Information (53). Antibiotic resistance genes were identified using the Comprehensive Antibiotic Resistance Database (CARD) (http://arpcard.mcmaster.ca) (54). *In silico* MLST typing was performed using LOCUST (55). The locations of insertion sequence elements in draft genomes were mapped using ISseeker (56). ISseeker finds partial IS matches at contig edges and maps the flanking sequence to a reference genome, in this case ACICU (GenBank accession number NC_010611.1). To define the structures of integrons, we began by identifying antibiotic resistance genes of interest (using a tBLASTn search with protein sequence queries of the draft genomes). Contigs containing these genes were then compared to a collection of previously described genomic islands and other mobile elements from A. baumannii, with particular attention being paid to well-characterized elements in reported genomes. Matching elements were then aligned to the draft genome, and an accounting was made of regions of identity and of difference. A set of nucleotide query sequences was then used to search the full set of genome sequences to identify all genomes with highly similar content. This back-and-forth strategy was used to refine the interpretation of the draft genome content. OC and K loci were mapped by BLAST of contigs from each genome to a database comprised of previously reported sequences (31, 32). An OC or K locus was considered a match if >98% of the locus was represented by sequences in the query genome. Pangenome analysis was performed using PanOCT (57) with the Cleveland genomes plus the 70 complete genomes from GenBank. Pairwise comparisons between the nucleotide sequences of all protein-coding genes were performed using an iterative approach to building the distance matrix. Fourteen groups were used in the first round of distance matrix construction, selected based on proximity on the phylogenetic tree. A gene presence/absence matrix was constructed, and the matrix was used to construct a neighbor-joining tree based on shared gene content.

### Phylogeny.

Genome sequences for 70 strains, representing complete genome sequences of all strains available in GenBank as of 28 June 2017, were included in the analysis to provide phylogenetic context for the Cleveland strains. In addition, all genomes that fall into the same clade as HUMC1 in the GenBank genomes division were downloaded and included in certain analyses of clade F to provide as broad a context as possible for the Cleveland genomes (250 additional genomes after removing 10 assemblies with more than 300 contigs). Single-nucleotide variants in the genome sequences were called using Parsnp (58), using the genome sequence of strain HUMC1 (15) as the reference. Three different genome groups were analyzed: (i) all the genomes described above, (ii) all GC2 genomes, and (iii) all clade F genomes. Parsnp calls SNVs in core genome regions, so the number of informative variants increases as the number of genes shared across the data set increases and as the genome diversity increases. To identify regions of recombination, SNVs were imputed onto the HUMC1 genome sequences using the FastaAlternateReferenceMaker feature of the Genome Analysis Toolkit (GATK) (59) to create the input sequences for Gubbins (60). Gubbins iteratively identifies regions of recombination based on the density of SNVs and simultaneously constructs a maximum likelihood tree based on the filtered polymorphisms using RAxML (61). For the all-GC2 tree in Fig. 1, 19,551 SNVs identified by Parsnp were reduced to 8,868 in nonrecombining regions by Gubbins. For the clade F tree in Fig. S2, 15,725 SNVs identified by Parsnp were reduced to 9,090 in nonrecombining regions.

### Data availability.

Data Set S1 in the supplemental material contains information about each strain, including GenBank accession numbers and antimicrobial susceptibility patterns, when available. Newly sequenced isolates have been registered at the NCBI under BioProject accession numbers PRJNA352251, PRJNA316619, and PRJNA271775.

## References

[1] Peleg AY, de Breij A, Adams MD, Cerqueira GM, Mocali S, Galardini M, Nibbering PH, Earl AM, Ward DV, Paterson DL, Seifert H, Dijkshoorn L 2012 The success of Acinetobacter species; genetic, metabolic and virulence attributes. PLoS One 7:e46984. doi:10.1371/journal.pone.0046984. 23144699 PMC3483291

[2] Potron A, Poirel L, Nordmann P 2015 Emerging broad-spectrum resistance in *Pseudomonas aeruginosa* and *Acinetobacter baumannii*: mechanisms and epidemiology. Int J Antimicrob Agents 45:568–585. doi:10.1016/j.ijantimicag.2015.03.001. 25857949

[3] Harding CM, Hennon SW, Feldman MF 2018 Uncovering the mechanisms of *Acinetobacter baumannii* virulence. Nat Rev Microbiol 16:91–102. doi:10.1038/nrmicro.2017.148. 29249812 PMC6571207

[4] Viehman JA, Nguyen MH, Doi Y 2014 Treatment options for carbapenem-resistant and extensively drug-resistant *Acinetobacter baumannii* infections. Drugs 74:1315–1333. doi:10.1007/s40265-014-0267-8. 25091170 PMC4258832

[5] Thom KA, Rock C, Jackson SS, Johnson JK, Srinivasan A, Magder LS, Roghmann MC, Bonomo RA, Harris AD 2017 Factors leading to transmission risk of *Acinetobacter baumannii*. Crit Care Med 45:e633–e639. doi:10.1097/CCM.0000000000002318. 28398924 PMC5474153

[6] Wright MS, Haft DH, Harkins DM, Perez F, Hujer KM, Bajaksouzian S, Benard MF, Jacobs MR, Bonomo RA, Adams MD 2014 New insights into dissemination and variation of the health care-associated pathogen *Acinetobacter baumannii* from genomic analysis. mBio 5:e00963-13. doi:10.1128/mBio.00963-13. 24449752 PMC3903280

[7] Nigro SJ, Post V, Hall RM 2011 Aminoglycoside resistance in multiply antibiotic-resistant *Acinetobacter baumannii* belonging to global clone 2 from Australian hospitals. J Antimicrob Chemother 66:1504–1509. doi:10.1093/jac/dkr163. 21586593

[8] Nemec A, Krizova L, Maixnerova M, Diancourt L, van der Reijden TJ, Brisse S, van den Broek P, Dijkshoorn L 2008 Emergence of carbapenem resistance in *Acinetobacter baumannii* in the Czech Republic is associated with the spread of multidrug-resistant strains of European clone II. J Antimicrob Chemother 62:484–489. doi:10.1093/jac/dkn205. 18477708

[9] Wright MS, Iovleva A, Jacobs MR, Bonomo RA, Adams MD 2016 Genome dynamics of multidrug-resistant *Acinetobacter baumannii* during infection and treatment. Genome Med 8:26. doi:10.1186/s13073-016-0279-y. 26939581 PMC4776386

[10] Diancourt L, Passet V, Nemec A, Dijkshoorn L, Brisse S 2010 The population structure of *Acinetobacter baumannii*: expanding multiresistant clones from an ancestral susceptible genetic pool. PLoS One 5:e10034. doi:10.1371/journal.pone.0010034. 20383326 PMC2850921

[11] Wallace L, Daugherty SC, Nagaraj S, Johnson JK, Harris AD, Rasko DA 2016 Use of comparative genomics to characterize the diversity of *Acinetobacter baumannii* surveillance isolates in a health care institution. Antimicrob Agents Chemother 60:5933–5941. doi:10.1128/AAC.00477-16. 27458211 PMC5038335

[12] Bartual SG, Seifert H, Hippler C, Luzon MA, Wisplinghoff H, Rodriguez-Valera F 2005 Development of a multilocus sequence typing scheme for characterization of clinical isolates of *Acinetobacter baumannii*. J Clin Microbiol 43:4382–4390. doi:10.1128/JCM.43.9.4382-4390.2005. 16145081 PMC1234098

[13] Mustapha MM, Li B, Pacey MP, Mettus RT, McElheny CL, Marshall CW, Ernst RK, Cooper VS, Doi Y 2018 Phylogenomics of colistin-susceptible and resistant XDR *Acinetobacter baumannii*. J Antimicrob Chemother 73:2952–2959. doi:10.1093/jac/dky290. 30124845 PMC6198730

[14] Fitzpatrick MA, Ozer EA, Hauser AR 2016 Utility of whole-genome sequencing in characterizing *Acinetobacter* epidemiology and analyzing hospital outbreaks. J Clin Microbiol 54:593–612. doi:10.1128/JCM.01818-15. 26699703 PMC4767972

[15] Luo G, Lin L, Ibrahim AS, Baquir B, Pantapalangkoor P, Bonomo RA, Doi Y, Adams MD, Russo TA, Spellberg B 2012 Active and passive immunization protects against lethal, extreme drug resistant-*Acinetobacter baumannii* infection. PLoS One 7:e29446. doi:10.1371/journal.pone.0029446. 22253723 PMC3254619

[16] Rolain JM, Roch A, Castanier M, Papazian L, Raoult D 2011 *Acinetobacter baumannii* resistant to colistin with impaired virulence: a case report from France. J Infect Dis 204:1146–1147. doi:10.1093/infdis/jir475. 21881132

[17] Rolain JM, Diene SM, Kempf M, Gimenez G, Robert C, Raoult D 2013 Real-time sequencing to decipher the molecular mechanism of resistance of a clinical pan-drug-resistant *Acinetobacter baumannii* isolate from Marseille, France. Antimicrob Agents Chemother 57:592–596. doi:10.1128/AAC.01314-12. 23070160 PMC3535948

[18] Buser GL, Cassidy PM, Cunningham MC, Rudin S, Hujer AM, Vega R, Furuno JP, Marshall SH, Higgins PG, Jacobs MR, Wright MS, Adams MD, Bonomo RA, Pfeiffer CD, Beldavs ZG 2017 Failure to communicate: transmission of extensively drug-resistant bla OXA-237-containing *Acinetobacter baumannii*—multiple facilities in Oregon, 2012-2014. Infect Control Hosp Epidemiol 38:1335–1341. doi:10.1017/ice.2017.189. 28870269 PMC5783543

[19] Hamidian M, Hall RM 2014 pACICU2 is a conjugative plasmid of *Acinetobacter* carrying the aminoglycoside resistance transposon Tn*aphA6*. J Antimicrob Chemother 69:1146–1148. doi:10.1093/jac/dkt488. 24335352

[20] Figueiredo S, Poirel L, Croize J, Recule C, Nordmann P 2009 *In vivo* selection of reduced susceptibility to carbapenems in *Acinetobacter baumannii* related to IS*Aba1*-mediated overexpression of the natural *bla*_OXA-66_ oxacillinase gene. Antimicrob Agents Chemother 53:2657–2659. doi:10.1128/AAC.01663-08. 19307373 PMC2687192

[21] Zander E, Chmielarczyk A, Heczko P, Seifert H, Higgins PG 2013 Conversion of OXA-66 into OXA-82 in clinical *Acinetobacter baumannii* isolates and association with altered carbapenem susceptibility. J Antimicrob Chemother 68:308–311. doi:10.1093/jac/dks382. 23014718

[22] Nigro SJ, Hall RM 2016 Structure and context of *Acinetobacter* transposons carrying the oxa23 carbapenemase gene. J Antimicrob Chemother 71:1135–1147. doi:10.1093/jac/dkv440. 26755496

[23] Mugnier P, Poirel L, Pitout M, Nordmann P 2008 Carbapenem-resistant and OXA-23-producing *Acinetobacter baumannii* isolates in the United Arab Emirates. Clin Microbiol Infect 14:879–882. doi:10.1111/j.1469-0691.2008.02056.x. 18844691

[24] Nigro SJ, Holt KE, Pickard D, Hall RM 2015 Carbapenem and amikacin resistance on a large conjugative *Acinetobacter baumannii* plasmid. J Antimicrob Chemother 70:1259–1261. doi:10.1093/jac/dku486. 25433005 PMC4356202

[25] Poirel L, Bonnin RA, Nordmann P 2011 Genetic basis of antibiotic resistance in pathogenic *Acinetobacter* species. IUBMB Life 63:1061–1067. doi:10.1002/iub.532. 21990280

[26] Chen CC, Lin YC, Sheng WH, Chen YC, Chang SC, Hsia KC, Liao MH, Li SY 2011 Genome sequence of a dominant, multidrug-resistant *Acinetobacter baumannii* strain, TCDC-AB0715. J Bacteriol 193:2361–2362. doi:10.1128/JB.00244-11. 21398540 PMC3133099

[27] Nigro SJ, Farrugia DN, Paulsen IT, Hall RM 2013 A novel family of genomic resistance islands, AbGRI2, contributing to aminoglycoside resistance in *Acinetobacter baumannii* isolates belonging to global clone 2. J Antimicrob Chemother 68:554–557. doi:10.1093/jac/dks459. 23169892

[28] Hamidian M, Hall RM 2011 AbaR4 replaces AbaR3 in a carbapenem-resistant *Acinetobacter baumannii* isolate belonging to global clone 1 from an Australian hospital. J Antimicrob Chemother 66:2484–2491. doi:10.1093/jac/dkr356. 21873287

[29] Fournier PE, Richet H 2006 The epidemiology and control of *Acinetobacter baumannii* in health care facilities. Clin Infect Dis 42:692–699. doi:10.1086/500202. 16447117

[30] Nigro SJ, Hall RM 2012 Tn6167, an antibiotic resistance island in an Australian carbapenem-resistant *Acinetobacter baumannii* GC2, ST92 isolate. J Antimicrob Chemother 67:1342–1346. doi:10.1093/jac/dks037. 22351684

[31] Kenyon JJ, Hall RM 2013 Variation in the complex carbohydrate biosynthesis loci of *Acinetobacter baumannii* genomes. PLoS One 8:e62160. doi:10.1371/journal.pone.0062160. 23614028 PMC3628348

[32] Kenyon JJ, Nigro SJ, Hall RM 2014 Variation in the OC locus of *Acinetobacter baumannii* genomes predicts extensive structural diversity in the lipooligosaccharide. PLoS One 9:e107833. doi:10.1371/journal.pone.0107833. 25247305 PMC4172580

[33] Kenyon JJ, Holt KE, Pickard D, Dougan G, Hall RM 2014 Insertions in the OCL1 locus of *Acinetobacter baumannii* lead to shortened lipooligosaccharides. Res Microbiol 165:472–475. doi:10.1016/j.resmic.2014.05.034. 24861001 PMC4110982

[34] Ronish LA, Lillehoj E, Fields JK, Sundberg EJ, Piepenbrink KH 2019 The structure of PilA from *Acinetobacter baumannii* AB5075 suggests a mechanism for functional specialization in *Acinetobacter* type IV pili. J Biol Chem 294:218–230. doi:10.1074/jbc.RA118.005814. 30413536 PMC6322890

[35] Marchaim D, Chopra T, Bogan C, Bheemreddy S, Sengstock D, Jagarlamudi R, Malani A, Lemanek L, Moshos J, Lephart PR, Ku K, Hasan A, Lee J, Khandker N, Blunden C, Geffert SF, Moody M, Hiro R, Wang Y, Ahmad F, Mohammadi T, Faruque O, Patel D, Pogue JM, Hayakawa K, Dhar S, Kaye KS 2012 The burden of multidrug-resistant organisms on tertiary hospitals posed by patients with recent stays in long-term acute care facilities. Am J Infect Control 40:760–765. doi:10.1016/j.ajic.2011.09.011. 22285709

[36] Mody L, Gibson KE, Horcher A, Prenovost K, McNamara SE, Foxman B, Kaye KS, Bradley S 2015 Prevalence of and risk factors for multidrug-resistant *Acinetobacter baumannii* colonization among high-risk nursing home residents. Infect Control Hosp Epidemiol 36:1155–1162. doi:10.1017/ice.2015.143. 26072936 PMC4626246

[37] Cerqueira GC, Earl AM, Ernst CM, Grad YH, Dekker JP, Feldgarden M, Chapman SB, Reis-Cunha JL, Shea TP, Young S, Zeng Q, Delaney ML, Kim D, Peterson EM, O’Brien TF, Ferraro MJ, Hooper DC, Huang SS, Kirby JE, Onderdonk AB, Birren BW, Hung DT, Cosimi LA, Wortman JR, Murphy CI, Hanage WP 2017 Multi-institute analysis of carbapenem resistance reveals remarkable diversity, unexplained mechanisms, and limited clonal outbreaks. Proc Natl Acad Sci U S A 114:1135–1140. doi:10.1073/pnas.1616248114. 28096418 PMC5293017

[38] Wong D, Nielsen TB, Bonomo RA, Pantapalangkoor P, Luna B, Spellberg B 2017 Clinical and pathophysiological overview of *Acinetobacter* infections: a century of challenges. Clin Microbiol Rev 30:409–447. doi:10.1128/CMR.00058-16. 27974412 PMC5217799

[39] Harding CM, Tracy EN, Carruthers MD, Rather PN, Actis LA, Munson RS Jr. 2013 *Acinetobacter baumannii* strain M2 produces type IV pili which play a role in natural transformation and twitching motility but not surface-associated motility. mBio 4:e00360-13. doi:10.1128/mBio.00360-13. 23919995 PMC3735195

[40] Piepenbrink KH, Lillehoj E, Harding CM, Labonte JW, Zuo X, Rapp CA, Munson RS Jr, Goldblum SE, Feldman MF, Gray JJ, Sundberg EJ 2016 Structural diversity in the type IV pili of multidrug-resistant *Acinetobacter*. J Biol Chem 291:22924–22935. doi:10.1074/jbc.M116.751099. 27634041 PMC5087714

[41] Miajlovic H, Smith SG 2014 Bacterial self-defence: how Escherichia coli evades serum killing. FEMS Microbiol Lett 354:1–9. doi:10.1111/1574-6968.12419. 24617921

[42] Chen L, Mathema B, Pitout JD, DeLeo FR, Kreiswirth BN 2014 Epidemic *Klebsiella pneumoniae* ST258 is a hybrid strain. mBio 5:e01355-14. doi:10.1128/mBio.01355-14. 24961694 PMC4073492

[43] Harding CM, Haurat MF, Vinogradov E, Feldman MF 2018 Distinct amino acid residues confer one of three UDP-sugar substrate specificities in *Acinetobacter baumannii* PglC phosphoglycosyltransferases. Glycobiology 28:522–533. doi:10.1093/glycob/cwy037. 29668902

[44] Halachev MR, Chan JZ, Constantinidou CI, Cumley N, Bradley C, Smith-Banks M, Oppenheim B, Pallen MJ 2014 Genomic epidemiology of a protracted hospital outbreak caused by multidrug-resistant *Acinetobacter baumannii* in Birmingham, England. Genome Med 6:70. doi:10.1186/s13073-014-0070-x. 25414729 PMC4237759

[45] Willems S, Kampmeier S, Bletz S, Kossow A, Kock R, Kipp F, Mellmann A 2016 Whole-genome sequencing elucidates epidemiology of nosocomial clusters of *Acinetobacter baumannii*. J Clin Microbiol 54:2391–2394. doi:10.1128/JCM.00721-16. 27358465 PMC5005500

[46] Lewis T, Loman NJ, Bingle L, Jumaa P, Weinstock GM, Mortiboy D, Pallen MJ 2010 High-throughput whole-genome sequencing to dissect the epidemiology of *Acinetobacter baumannii* isolates from a hospital outbreak. J Hosp Infect 75:37–41. doi:10.1016/j.jhin.2010.01.012. 20299126

[47] Schultz MB, Pham Thanh D, Tran Do Hoan N, Wick RR, Ingle DJ, Hawkey J, Edwards DJ, Kenyon JJ, Phu Huong Lan N, Campbell JI, Thwaites G, Thi Khanh Nhu N, Hall RM, Fournier-Level A, Baker S, Holt KE 2016 Repeated local emergence of carbapenem-resistant Acinetobacter baumannii in a single hospital ward. Microb Genom 2:e000050. doi:10.1099/mgen.0.000050. 28348846 PMC5320574

[48] Wendel AF, Malecki M, Otchwemah R, Tellez-Castillo CJ, Sakka SG, Mattner F 2018 One-year molecular surveillance of carbapenem-susceptible *A. baumannii* on a German intensive care unit: diversity or clonality. Antimicrob Resist Infect Control 7:145. doi:10.1186/s13756-018-0436-8. 30505434 PMC6260569

[49] Grana-Miraglia L, Lozano LF, Velazquez C, Volkow-Fernandez P, Perez-Oseguera A, Cevallos MA, Castillo-Ramirez S 2017 Rapid gene turnover as a significant source of genetic variation in a recently seeded population of a healthcare-associated pathogen. Front Microbiol 8:1817. doi:10.3389/fmicb.2017.01817. 28979253 PMC5611417

[50] Feng Y, Ruan Z, Shu J, Chen CL, Chiu CH 2016 A glimpse into evolution and dissemination of multidrug-resistant *Acinetobacter baumannii* isolates in East Asia: a comparative genomics study. Sci Rep 6:24342. doi:10.1038/srep24342. 27072398 PMC4829828

[51] Koser CU, Fraser LJ, Ioannou A, Becq J, Ellington MJ, Holden MT, Reuter S, Torok ME, Bentley SD, Parkhill J, Gormley NA, Smith GP, Peacock SJ 2014 Rapid single-colony whole-genome sequencing of bacterial pathogens. J Antimicrob Chemother 69:1275–1281. doi:10.1093/jac/dkt494. 24370932 PMC3977605

[52] Zerbino DR, Birney E 2008 Velvet: algorithms for de novo short read assembly using de Bruijn graphs. Genome Res 18:821–829. doi:10.1101/gr.074492.107. 18349386 PMC2336801

[53] Tatusova T, DiCuccio M, Badretdin A, Chetvernin V, Ciufo S, Li W 2013 Prokaryotic genome annotation pipeline, p 173–186. *In* Beck J, Benson D, Coleman J, Hoeppner M, Johnson M, Maglott D, Mizrachi I, Morris R, Ostell J, Pruitt K, Rubinstein W, Sayers E, Sirotkin K, Tatusova T (ed), NCBI handbook, 2nd ed National Center for Biotechnology Information, Bethesda, MD https://www.ncbi.nlm.nih.gov/books/NBK174280.

[54] Jia B, Raphenya AR, Alcock B, Waglechner N, Guo P, Tsang KK, Lago BA, Dave BM, Pereira S, Sharma AN, Doshi S, Courtot M, Lo R, Williams LE, Frye JG, Elsayegh T, Sardar D, Westman EL, Pawlowski AC, Johnson TA, Brinkman FS, Wright GD, McArthur AG 2017 CARD 2017: expansion and model-centric curation of the comprehensive antibiotic resistance database. Nucleic Acids Res 45:D566–D573. doi:10.1093/nar/gkw1004. 27789705 PMC5210516

[55] Brinkac LM, Beck E, Inman J, Venepally P, Fouts DE, Sutton G 2017 LOCUST: a custom sequence locus typer for classifying microbial isolates. Bioinformatics 33:1725–1726. doi:10.1093/bioinformatics/btx045. 28130240 PMC5860141

[56] Adams MD, Bishop B, Wright MS 2016 Quantitative assessment of insertion sequence impact on bacterial genome architecture. Microb Genom 2:e000062. doi:10.1099/mgen.0.000062. 28348858 PMC5343135

[57] Fouts DE, Brinkac L, Beck E, Inman J, Sutton G 2012 PanOCT: automated clustering of orthologs using conserved gene neighborhood for pan-genomic analysis of bacterial strains and closely related species. Nucleic Acids Res 40:e172. doi:10.1093/nar/gks757. 22904089 PMC3526259

[58] Treangen TJ, Ondov BD, Koren S, Phillippy AM 2014 The Harvest suite for rapid core-genome alignment and visualization of thousands of intraspecific microbial genomes. Genome Biol 15:524. doi:10.1186/s13059-014-0524-x. 25410596 PMC4262987

[59] McKenna A, Hanna M, Banks E, Sivachenko A, Cibulskis K, Kernytsky A, Garimella K, Altshuler D, Gabriel S, Daly M, DePristo MA 2010 The Genome Analysis Toolkit: a MapReduce framework for analyzing next-generation DNA sequencing data. Genome Res 20:1297–1303. doi:10.1101/gr.107524.110. 20644199 PMC2928508

[60] Croucher NJ, Page AJ, Connor TR, Delaney AJ, Keane JA, Bentley SD, Parkhill J, Harris SR 2015 Rapid phylogenetic analysis of large samples of recombinant bacterial whole genome sequences using Gubbins. Nucleic Acids Res 43:e15. doi:10.1093/nar/gku1196. 25414349 PMC4330336

[61] Stamatakis A 2014 RAxML version 8: a tool for phylogenetic analysis and post-analysis of large phylogenies. Bioinformatics 30:1312–1313. doi:10.1093/bioinformatics/btu033. 24451623 PMC3998144

