# Supplementary figures and images for: Rapid Replacement of Acinetobacter baumannii Strains Accompanied by Changes in Lipooligosaccharide Loci and Resistance Gene Repertoire
Source: mBio. 2019 Mar 26;10(2):e00356-19. doi: 10.1128/mBio.00356-19 (PMC6437055; doi:10.1128/mBio.00356-19)

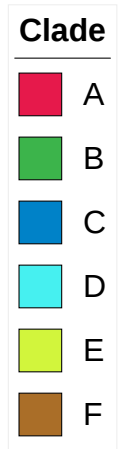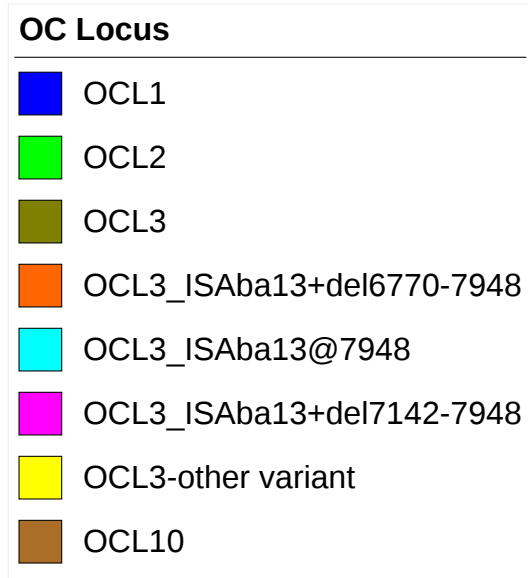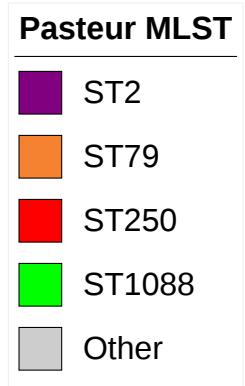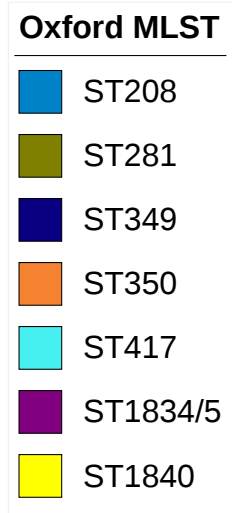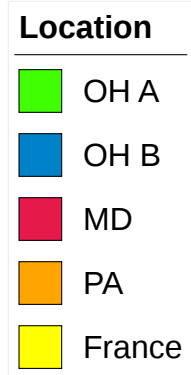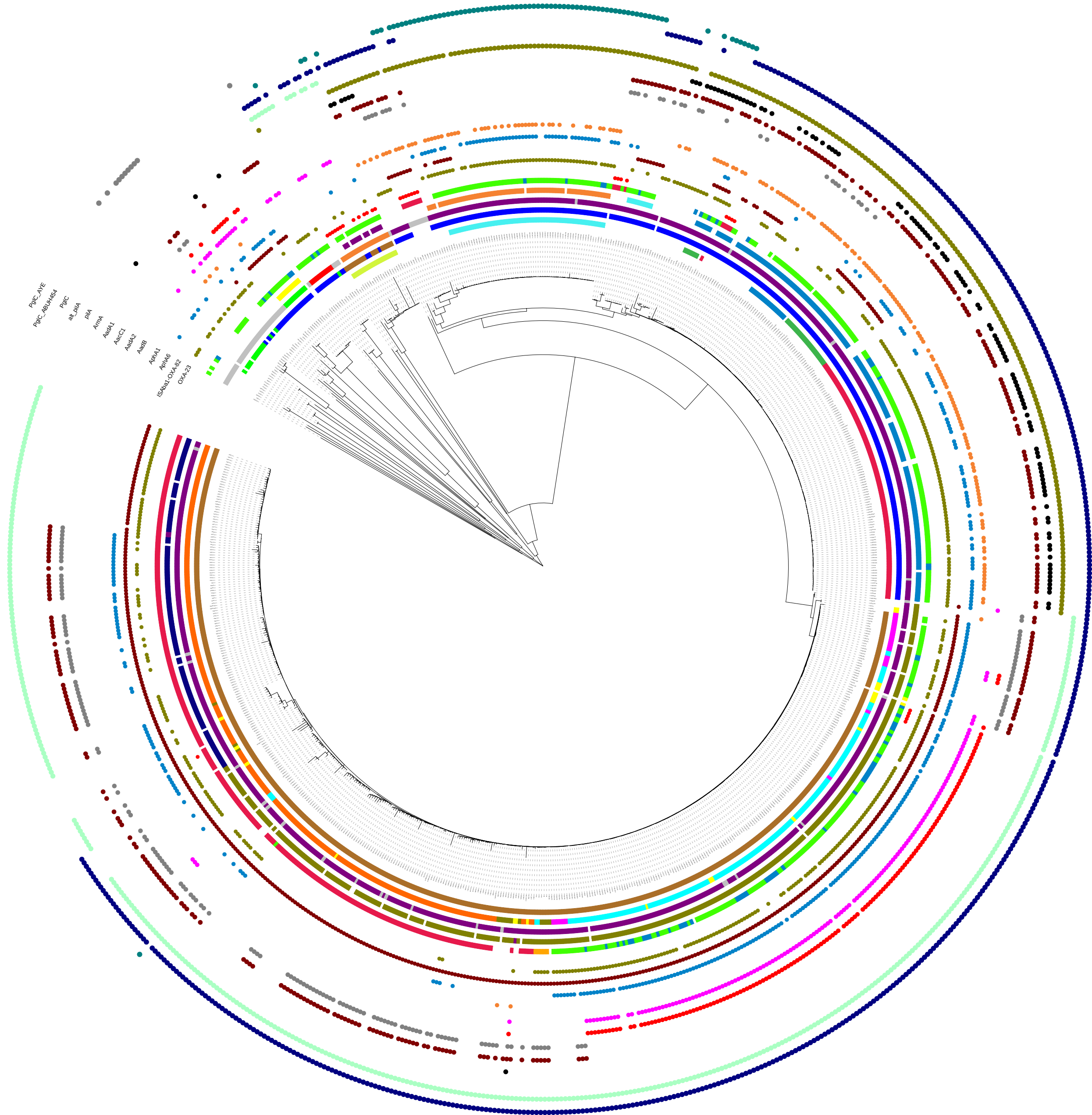

Supplement: FIG S1 [file mBio.00356-19-sf001.pdf]

(A)

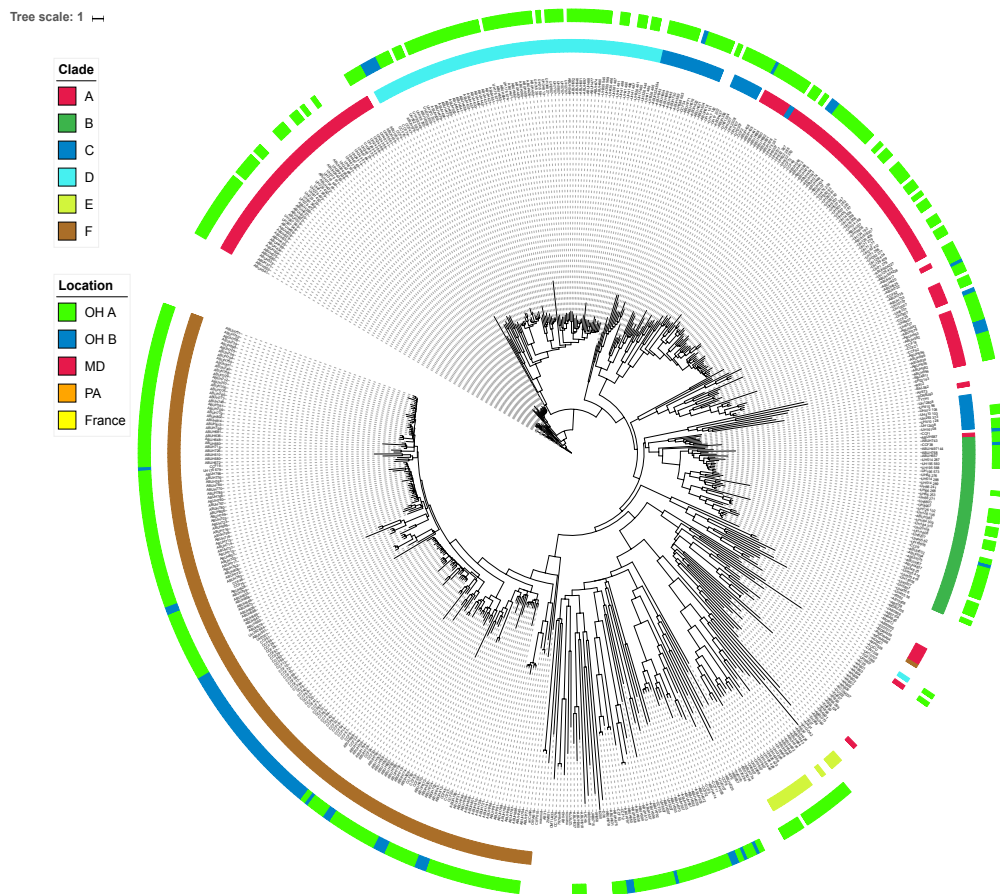

(B)

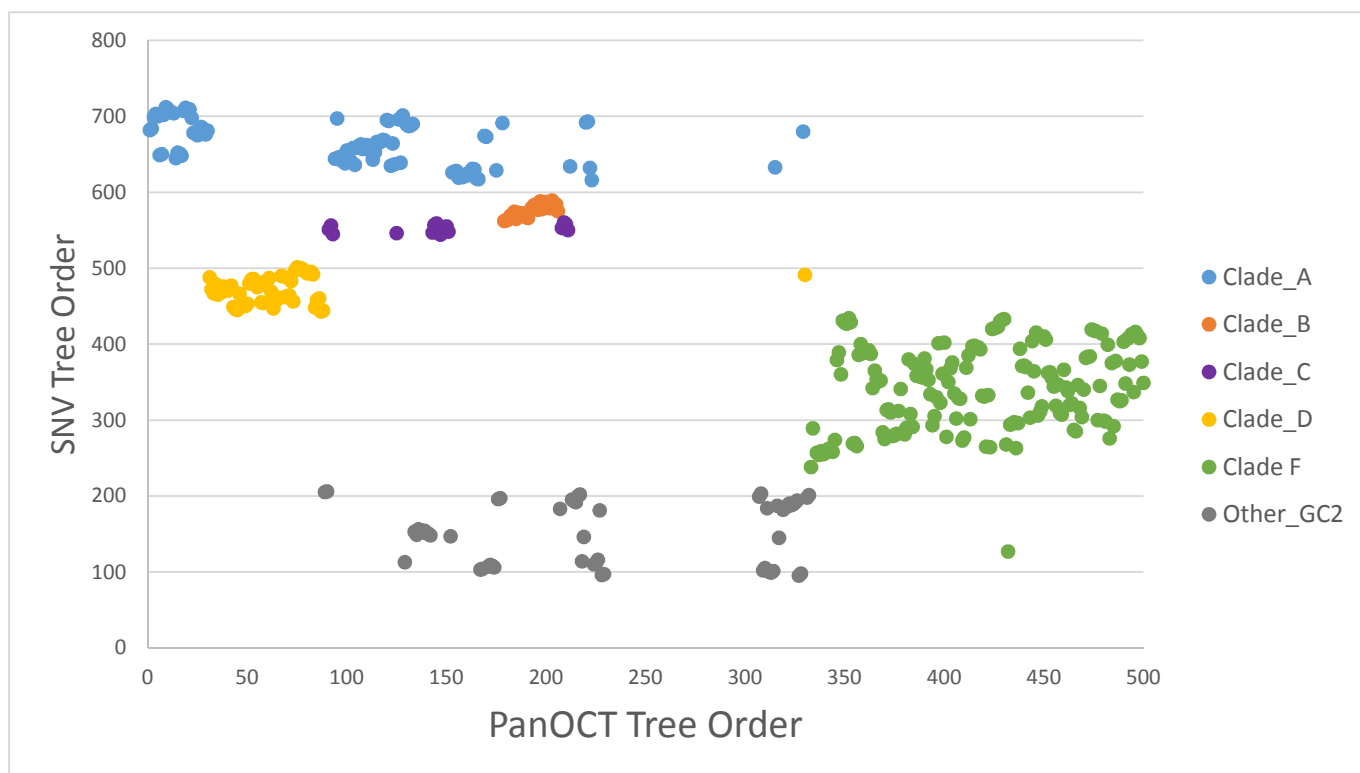

Supplement: FIG S3 [file mBio.00356-19-sf003.pdf]
