# Supplementary material for: Rapid Replacement of Acinetobacter baumannii Strains Accompanied by Changes in Lipooligosaccharide Loci and Resistance Gene Repertoire
Source: mBio. 2019 Mar 26;10(2):e00356-19. doi: 10.1128/mBio.00356-19 (PMC6437055; doi:10.1128/mBio.00356-19)
Supplement: FIG S2 [file mBio.00356-19-sf002.pdf]

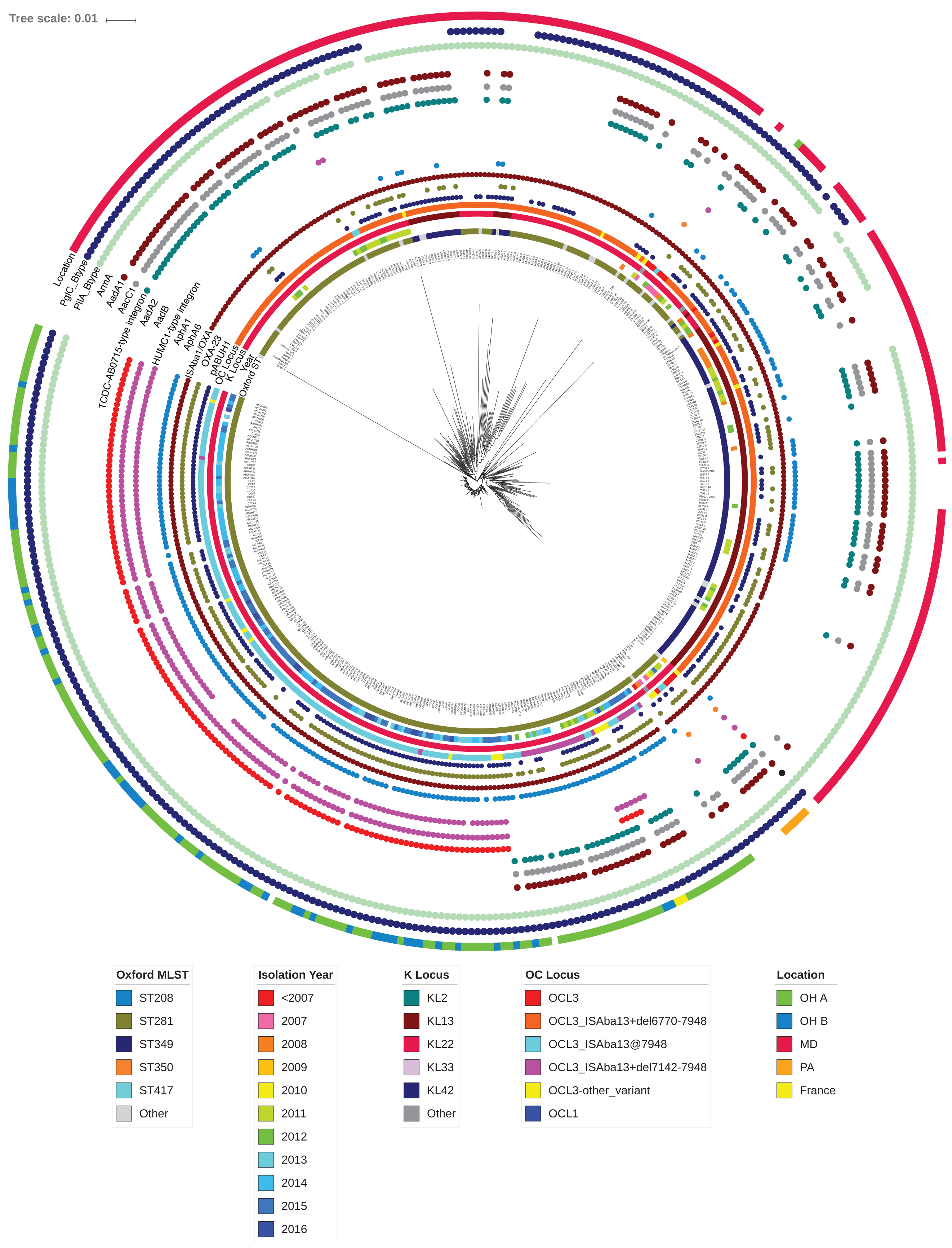

Tree scale: 0.01
Location
PglC_Btype
PilA_Btype
ArmA
AadA1
AacC1
AadA2
AadB
TODC-AB0715-type integron
HUMC1-type integron
AphA1
AphA6
ISAba1/OXA
OXA-23
pABUH1
OC Locus
K Locus
Year
Oxford ST
Oxford MLST
ST208
ST281
ST349
ST350
ST417
Other
Isolation Year
<2007
2007
2008
2009
2010
2011
2012
2013
2014
2015
2016
K Locus
KL2
KL13
KL22
KL33
KL42
Other
OC Locus
OCL3
OCL3_ISAba13+del6770-7948
OCL3_ISAba13@7948
OCL3_ISAba13+del7142-7948
OCL3-other_variant
OCL1
Location
OH A
OH B
MD
PA
France
